# Supplementary material for: Genomics of Staphylococcus aureus Strains Isolated from Infectious and Non-Infectious Ocular Conditions
Source: Antibiotics (Basel). 2022 Jul 27;11(8):1011. doi: 10.3390/antibiotics11081011 (PMC9405196; doi:10.3390/antibiotics11081011)
Supplement: Supplementary file 1 [file antibiotics-11-01011-s001.zip › antibiotics-1826497-supplementary.pdf]

Supplementary Table S1. Genomic features of *S. aureus* non-ocular isolates

S. aureus isolates	Accession number	Source	MLST	GC%	Contigs	Core genes	Shell genes	Pan/total genes
**M121**	GCA_001021875.1	Nasal	ST8	32.9	1	2494	388	2882
**USA300_SUR17**	GCA_002000725.1	Nasal	ST8	32.8	1	2518	360	2878
**Mu50**	GCA_000009665.1	Skin/Soft Tissue	ST5	32.9	1	2324	684	3008
**JKd6159**	GCA_000144955.1	Skin/Soft Tissue	ST93	32.8	1	2190	875	3065
**2148.N**	GCA_001717665.2	Nasal	ST72	32.8	1	2288	585	2873
**CA12**	GCA_001045795.2	Blood	ST8	32.7	1	2482	321	2803
**FDAARGOS_159**	GCA_001558795.2	Skin/Soft Tissue	ST8	32.9	1	2330	591	2921
**JH9**	GCA_000016805.1	Blood	ST105	32.9	1	2190	875	3065
**USA_ISMMs1**	GCA_000568455.1	Heart	ST8	32.8	1	2512	360	2872
**USA300_FPR3757**	GCA_000013465.1	Joint	ST8	32.8	1	2479	356	2835
